# Supplementary material for: TRIM24 is an oncogenic transcriptional co-activator of STAT3 in glioblastoma
Source: Nat Commun. 2017 Nov 13;8:1454. doi: 10.1038/s41467-017-01731-w (PMC5682287; doi:10.1038/s41467-017-01731-w)
Supplement: Supplementary file 1 — Supplementary Information [file 41467_2017_1731_MOESM1_ESM.pdf]

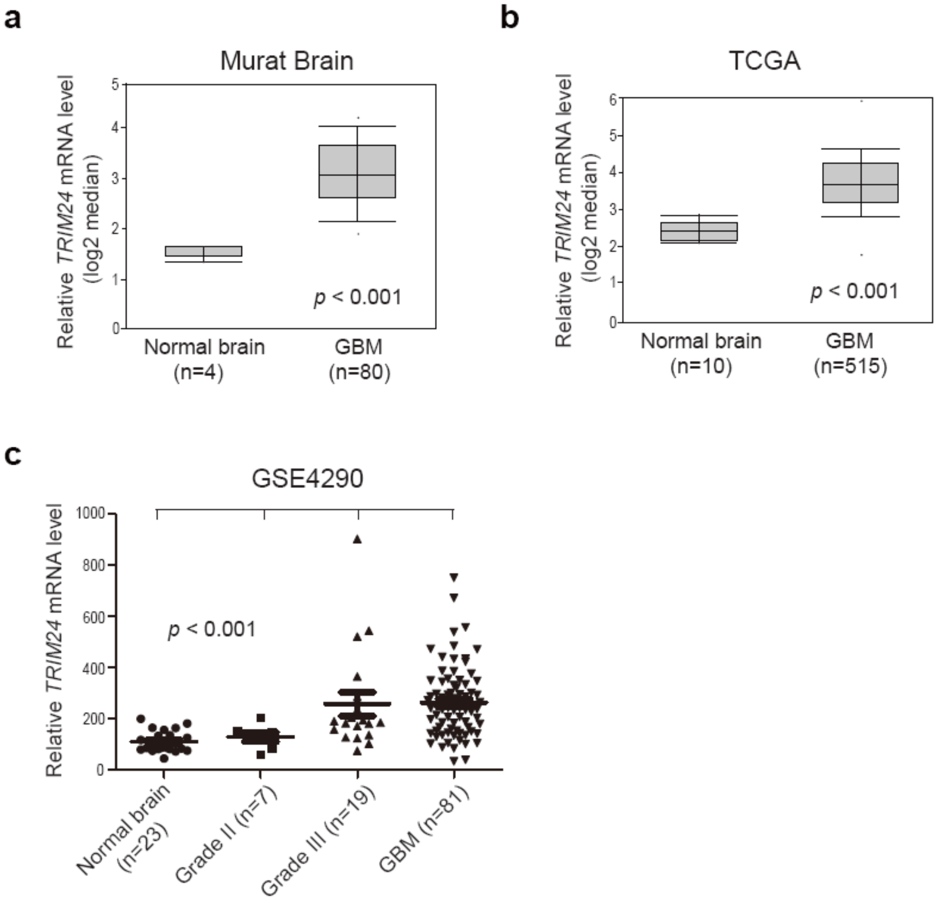

Supplementary Figure 1. TRIM24 is highly expressed and amplified in GBM. (**a**) Oncomine analysis of expression levels of *TRIM24* mRNA in normal brains and GBM from Murat Brain dataset. (**b**) Oncomine analysis of expression levels of *TRIM24* mRNA in normal brains and GBM from TCGA dataset. (**c**) Analysis of expression levels of *TRIM24* mRNA in normal brain, astrocyte Grade II-III, and GBM from GSE4290 dataset. *P* values were calculated by using paired two-way Student's *t*-test (**a** and **b**) and one-way analysis of variance with Newman-Keuls post-hoc test (**c**). In a and b, box plots with whiskers from minimum to maximum.

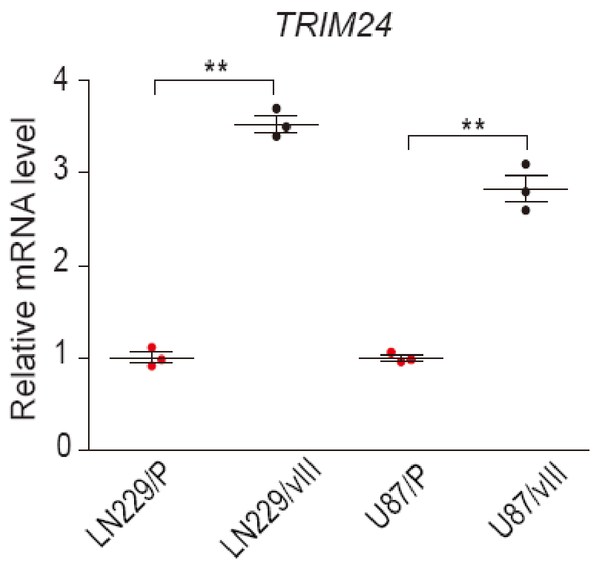

Supplementary Figure 2. qRT-PCR analysis of effects of EGFRvIII on TRIM24 mRNA expression in LN229 and U87 GBM cells. Error bars, s.d. Data represents two independent experiments with similar results. ***P* < 0.01, paired two-way Student's *t*-test.

a

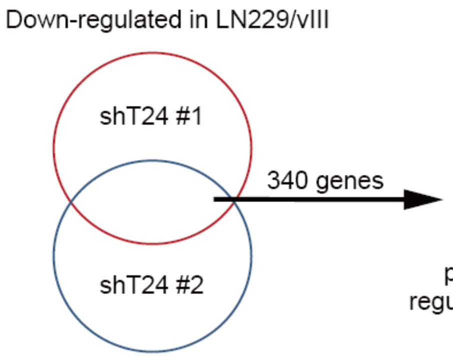

b

shT24-down-regulated pathways	% genes in list	p-value
cell division	8.8	4.18E-12
DNA replication	6.0	5.28E-11
G1/S transition of mitotic cell cycle	4.4	3.33E-09
mitotic nuclear division	6.5	4.11E-09
DNA replication initiation	2.7	6.52E-08
Cell proliferation	6.2	9.59E-06
regulation of cell cycle	2.9	3.70E-04
positive regulation of cell proliferation	5.3	0.006
regulation of transcription involved in G1	1.2	0.008
G2/M transition of mitotic cell cycle	2.4	0.012

Supplementary Figure 3. TRIM24 activates cell proliferation pathways in GBM cells. (**a**) Venn diagram of the comparison of RNA-Seq experiments between LN229/vIII with shT24 #1 and LN229/vIII with shT24 #2. (**b**) Gene ontology (GO) analysis indicates that TRIM24 shRNA-downregulated cell proliferation pathways in LN229 GBM cells.

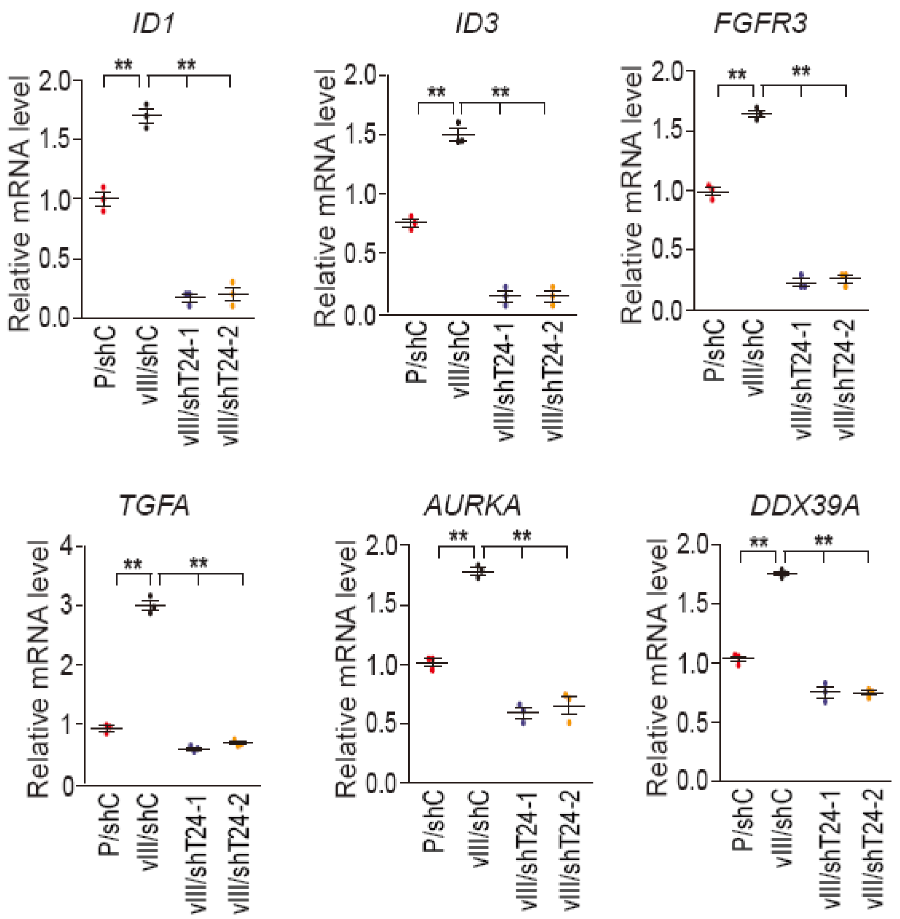

Supplementary Figure 4. qRT-PCR analysis of *ID1*, *ID3*, *FGFR3*, *TGFA*, *AURKA* and *DDX39A* expression in LN229 with shC (P/shC), vIII/shC, vIII/shT24-1 and vIII/shT24-2. *ACTN* was used as a control. Error bars, s.d. Data represent three independent experiments with similar results. $^{**}P < 0.01$, paired two-way Student's *t*-test.

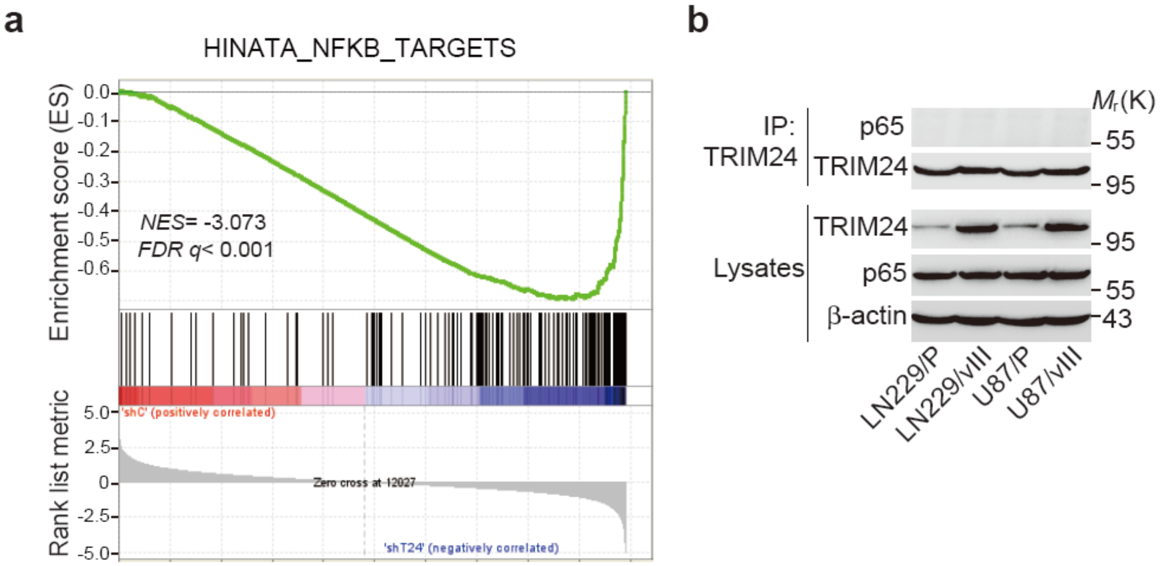

Supplementary Figure 5. NF-κB signaling pathway is regulated by TRIM24 in glioma cells. (**a**) Gene set enrichment analysis of NF-κB target genes[1] using ranked gene expression changes in *TRIM24*-knockdown LN229/EGFRvIII cells versus control cells. *NES,* normalized enrichment score. (**b**) IP and WB analyses of TRIM24 and p65 expression and binding in LN229 and U87 GBM cells with, or without EGFRvIII overexpression. Data are representative of three independent experiments with similar results.

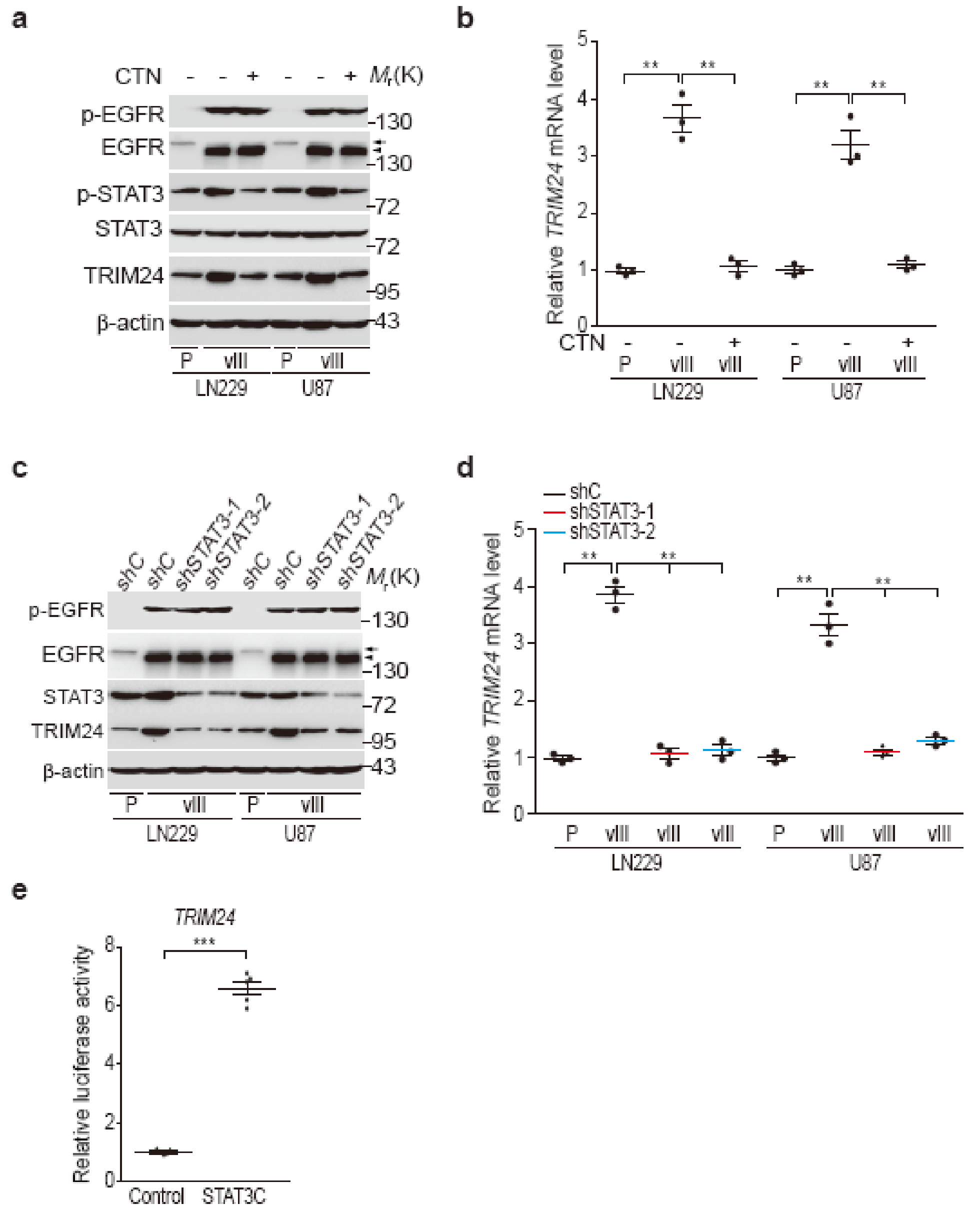

Supplementary Figure 6. STAT3 regulates *TRIM24* transcription. (**a**) WB analyses of STAT3 inhibitor cryptotanshinone (CTN) treatment on EGFRvIII-regulated TRIM24 protein expression in LN229 and U87 GBM cells. Cells were treated with or without cryptotanshinone (2 μM) for 24 h. (**b**) qRT-PCR analysis of CTN treatment on EGFRvIII-regulated *TRIM24* mRNA expression. Western blotting assays of effect of *STAT3* knockdown with two different shRNAs (shSTAT3-1 and shSTAT3-2) or a control shRNA on EGFRvIII-regulated TRIM24 expression in LN229 and U87 GBM cells. (**c**) WB analyses of *STAT3* knockdown with two different shRNAs (shSTAT3-1 and shSTAT3-2) or a control shRNA in LN229 and U87 GBM cells with, or without EGFRvIII overexpression. (**d**) qRT-PCR analysis of *STAT3* knockdown on EGFRvIII-regulated *TRIM24* mRNA expression. (**e**) Overexpression of STAT3C (constitutively active STAT3) activates *TRIM24* transcription in U87 GBM cells. U87 cells were transfected with TRIM24 reporter with or without STAT3-CA. Error bars, s.d. Data represent two or three independent experiments with similar results. ***P* < 0.01, ****P* < 0.001, paired two-way Student's *t*-test.

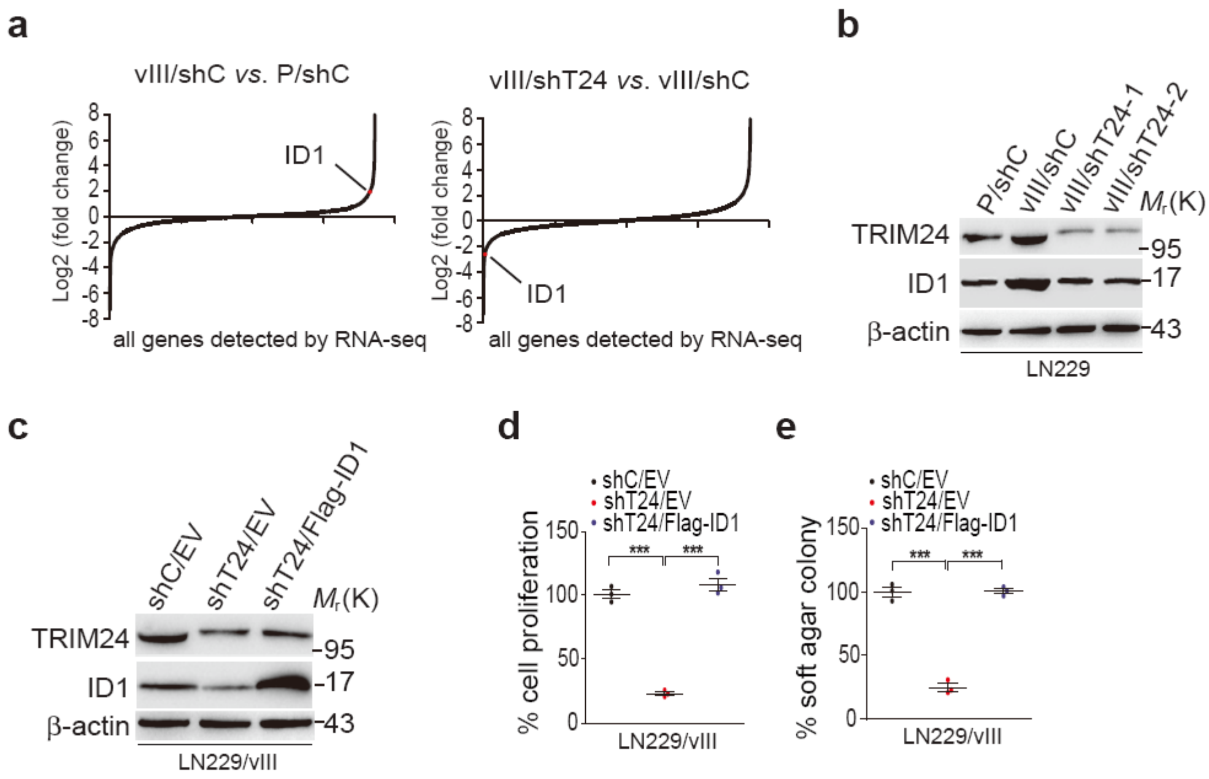

Supplementary Figure 7. TRIM24 and EGFRvIII co-upregulates ID1 in GBM cells. (**a**) Waterfall plots show gene-expression changes among LN229/P/shC, LN229/vIII/shC, LN229/vIII/shT24 #1 and LN229/vIII/shT24 #2 GBM cells. (**b**) WB assays of effect of knockdown of TRIM24 on EGFRvIII-driven ID1 expression in LN229 GBM cells. β-actin was used as a control. (**c**) WB assays of over-expression of Flag-ID1 in LN229/vIII/shT24 GBM cells. (**d** and **e**) Effects of over-expression of Flag-ID1 on EGFRvIII-driven cell proliferation (**d**) and colony formation in soft agar (**e**). Error bars, s.d. Data represent two or three independent experiments with similar results. ***$P < 0.001$, paired two-way Student's *t*-test.

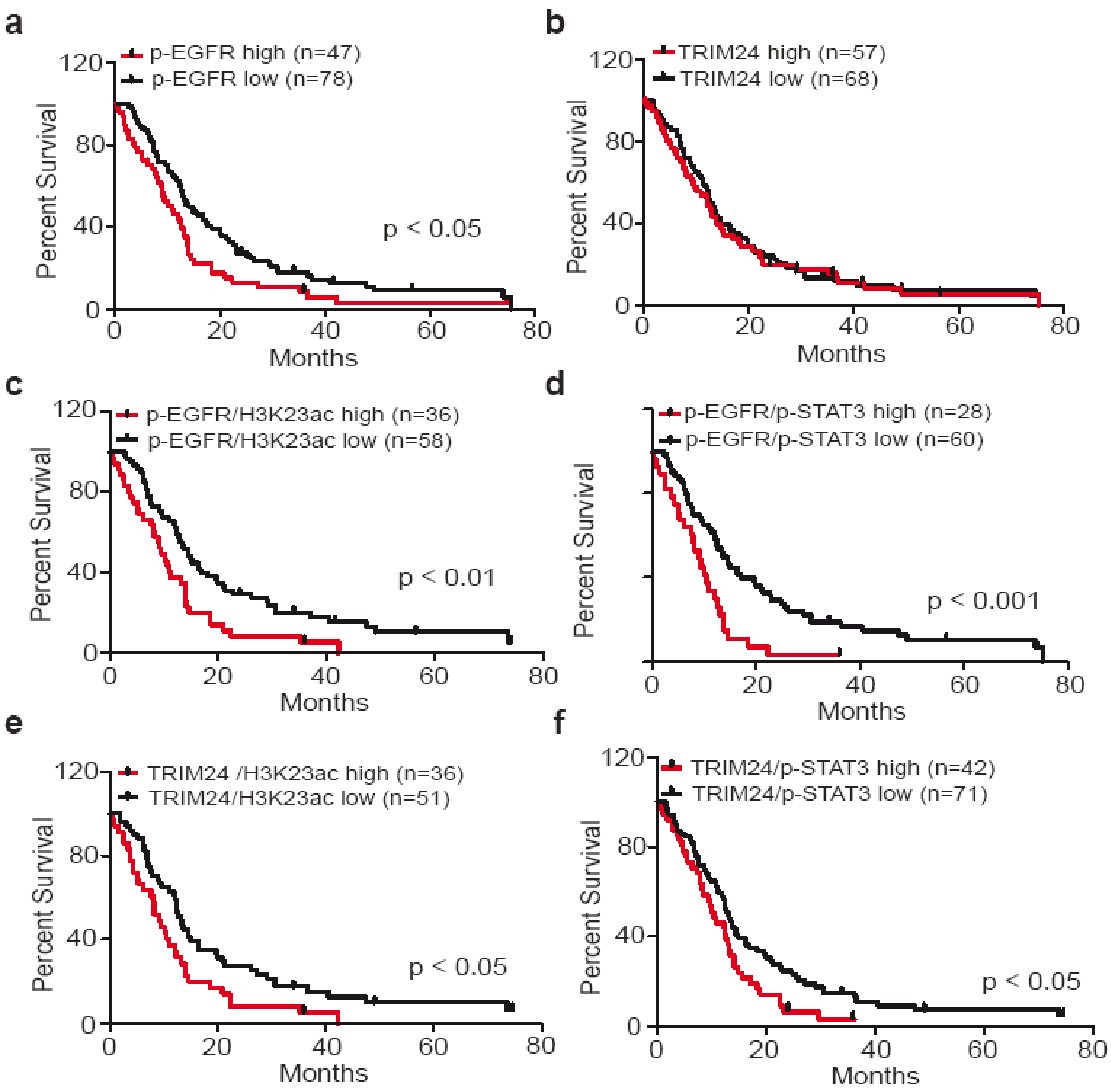

Supplementary Figure 8. Co-expression of TRIM24 with p-EGFR, H3K23ac, or p-STAT3 correlates with worse survival of patients with GBM. Kaplan-Meier analyses of patient's survial in IHC-stained GBM specimens showed in Fig. 9A: (**a**) high expression levels of p-EGFR tumors (red line) versus low p-EGFR tumor (black line). Median surval (in months): high, 10.8, low, 13.5. (**b**) high expression levels of TRIM24 tumors (red line) versus low TRIM24 tumors (black line). (**c**) co-expression of high p-EGFR/H3K23ac tumors (red line) versus low p-EGFR/H3K23ac-expressing tumors (black line). Median surval (in months): high, 9.3, low, 14.5. (**d**) co-expression of high p-EGFR/p-STAT3 tumors (red line) versus low p-EGFR/p-STAT3-expressing tumors (black line). Median surval (in months): high, 9.0, low, 13.5. (**e**) co-expression of high TRIM24/H3K23ac tumors (red line) versus low TRIM24/H3K23ac-expressing tumors (black line). Median surval (in months): high, 10.0, low, 13.1. (**f**) co-expression of high TRIM24/p-STAT3 tumors (red line) versus low TRIM24/p-STAT3-expressing tumors (black line). Median surval (in months): high, 10.4, low, 13.1. *P* values were calculated by using log-rank test. Black bars, censored data.

Full unedited gels for Figure 1

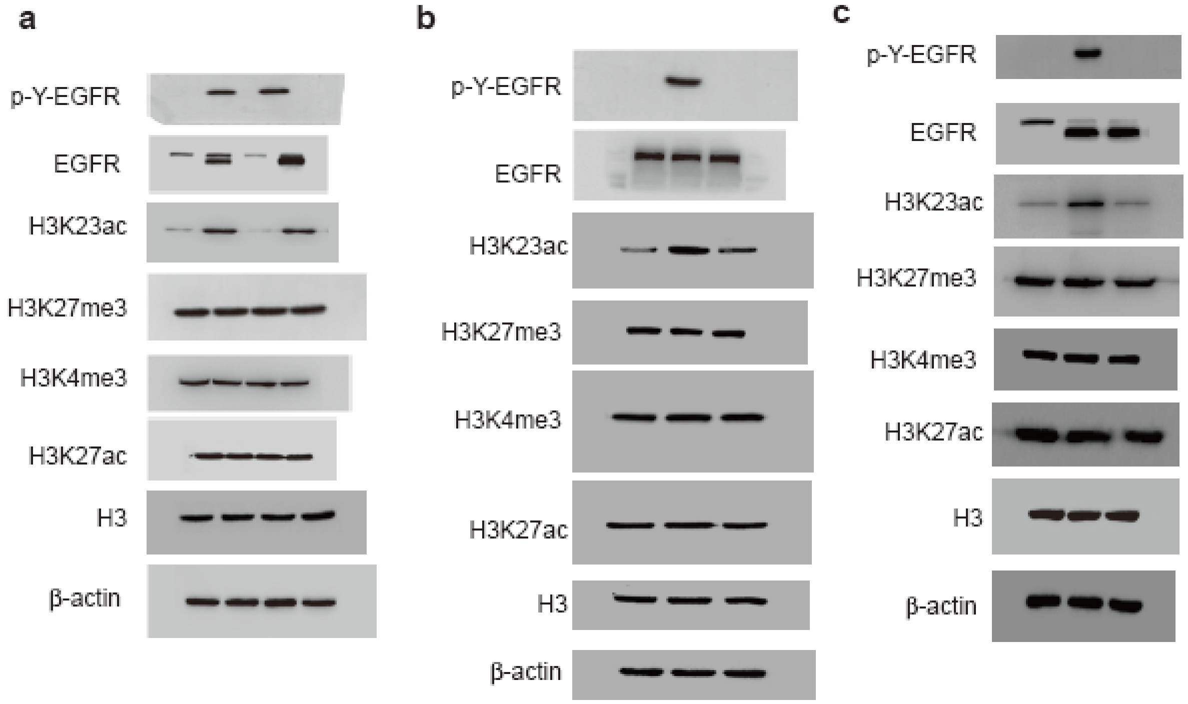

Full unedited gels for Figure 2

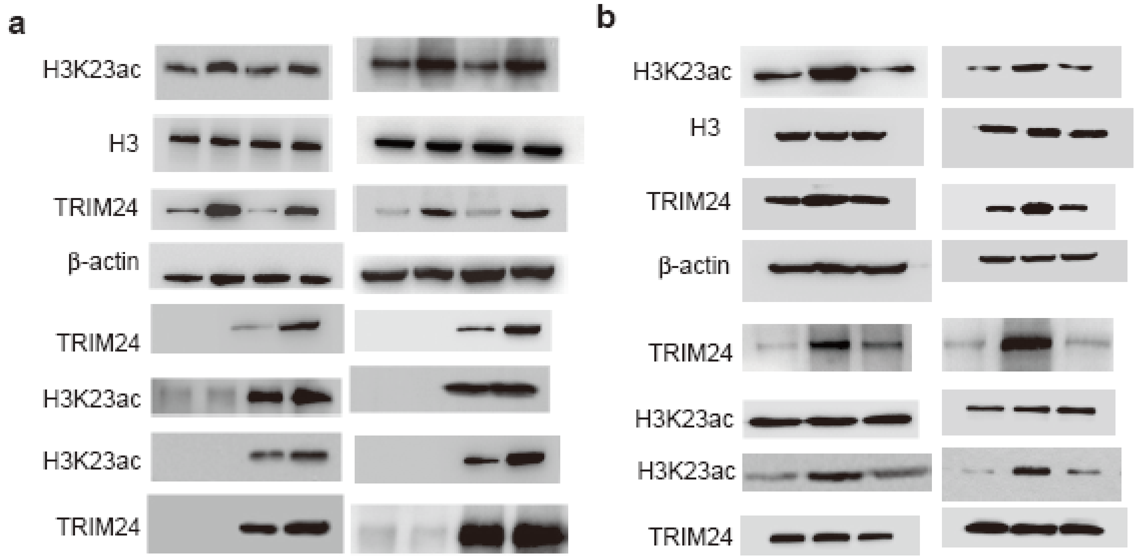

Full unedited gels for Figure 3

a

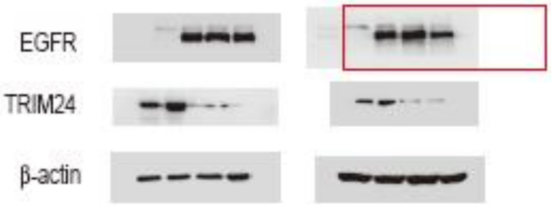

Full unedited gels for Figure 4

b

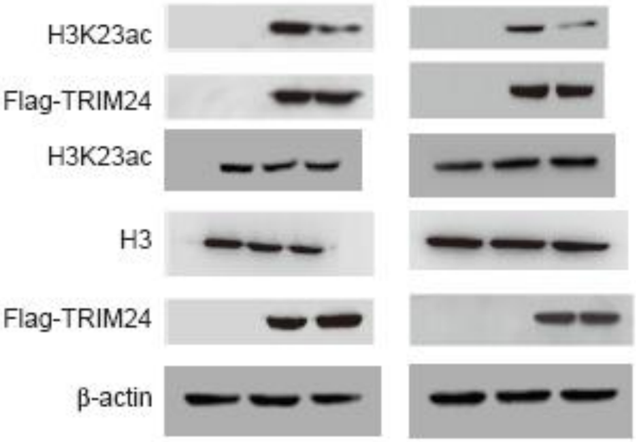

Full unedited gels for Figure 6

b

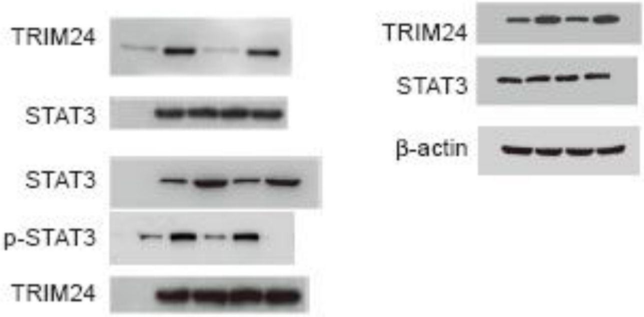

Full unedited gels for Figure 7

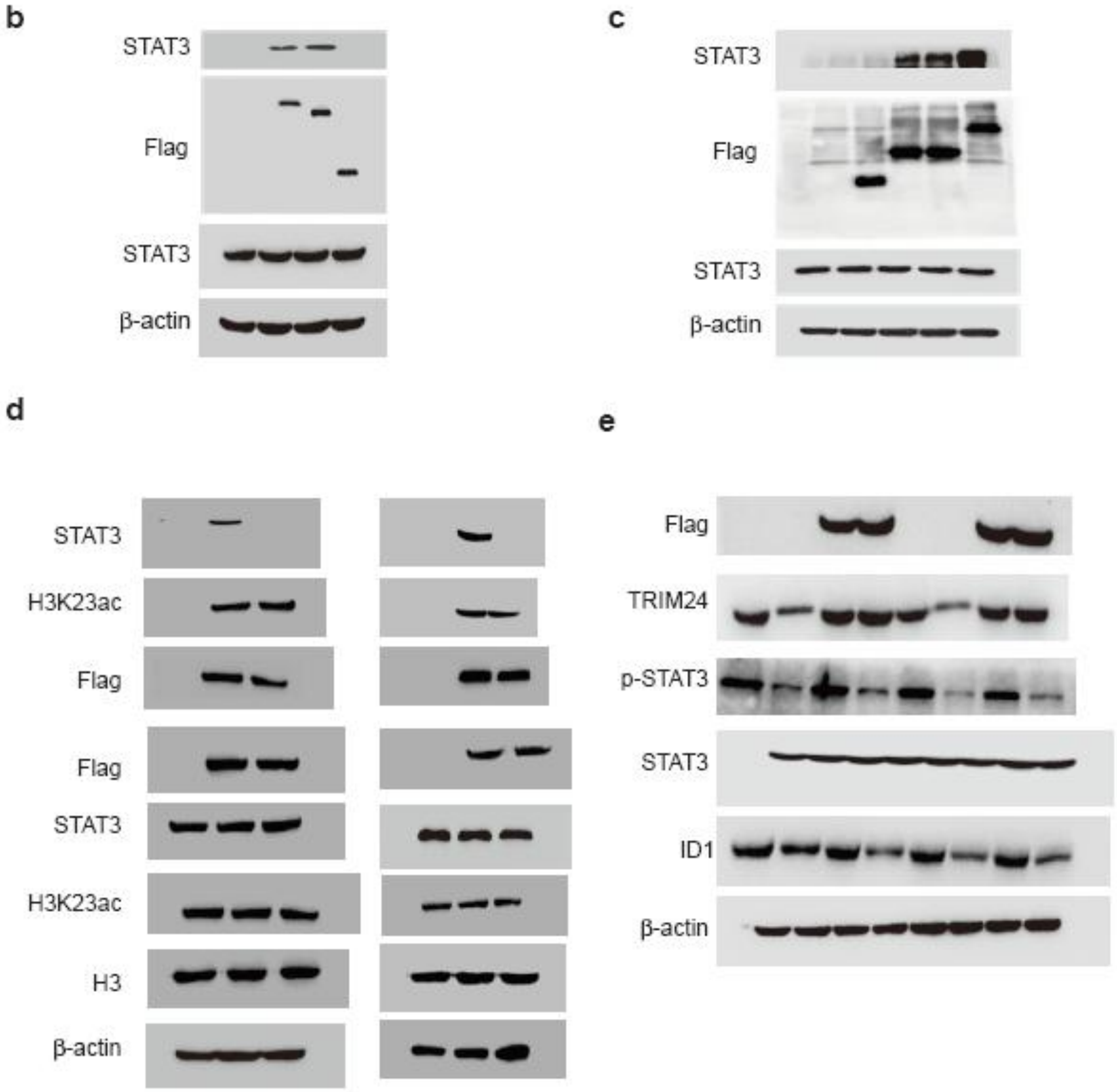

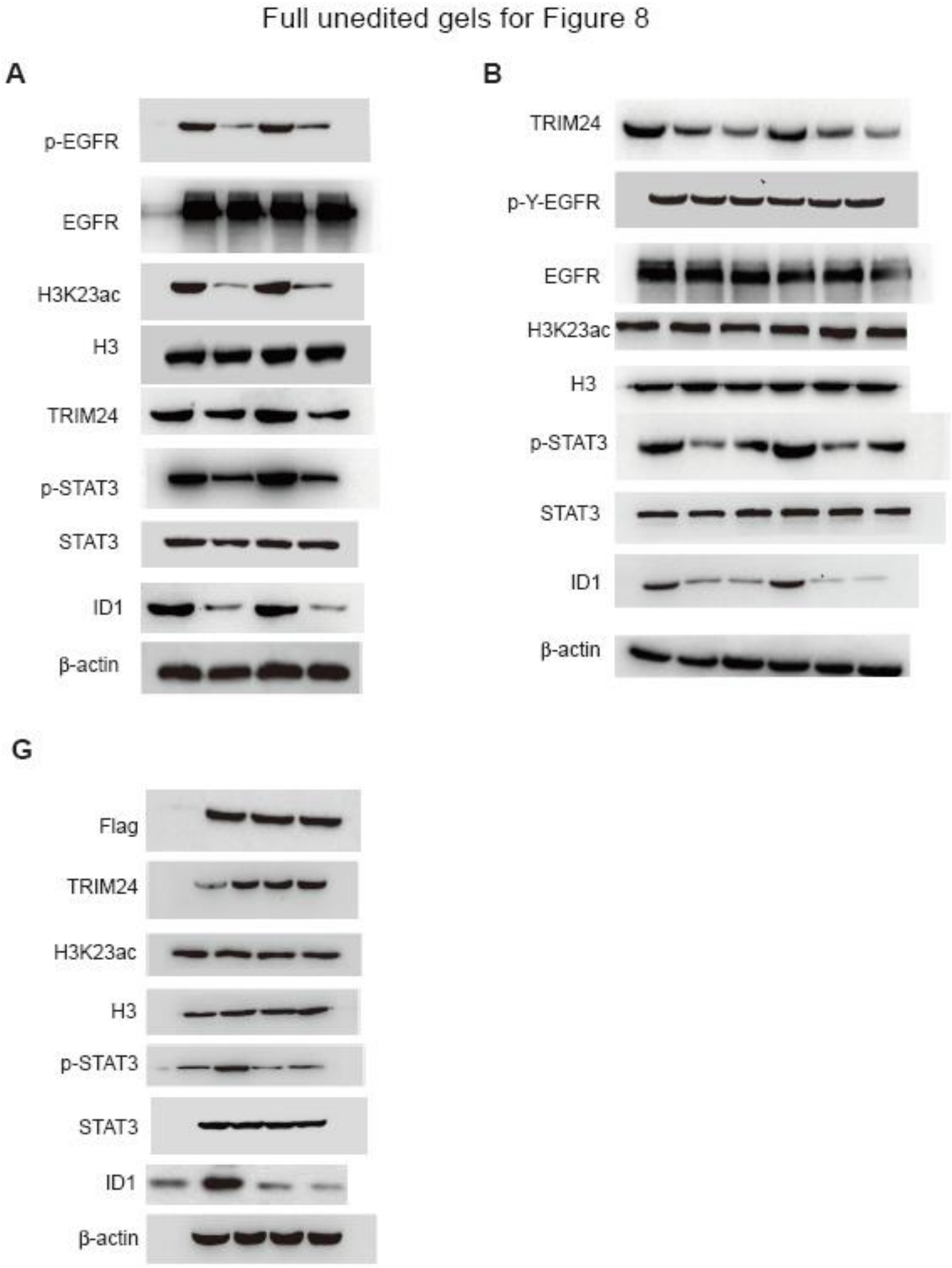

Supplementary Figure 9. Full unedited gels.

Supplementary Table 1. Spearman's rank correlation analysis of expression level of p-EGFR, TRIM24, H3K23ac and p-STAT3 in human clinical GBM specimens by IHC staining.

r	p-EGFR	TRIM24	H3K23ac	p-STAT3
p-EGFR	1.0*	0.4*	0.6*	0.3*
TRIM24	0.4*	1.0*	0.3*	0.8*
H3K23ac	0.6*	0.3*	1.0*	0.3*
p-STAT3	0.3*	0.8*	0.3*	1.0*

Note: *, $P < 0.001$, paired two-way Student's *t*-test.

References

1. Hinata K, Gervin AM, Jennifer Zhang Y, Khavari PA. Divergent gene regulation and growth effects by NF-kappa B in epithelial and mesenchymal cells of human skin. *Oncogene* **22**, 1955-1964 (2003).
